# Achieve Location Privacy-Preserving Range Query in Vehicular Sensing

**DOI:** 10.3390/s17081829

**Published:** 2017-08-08

**Authors:** Qinglei Kong, Rongxing Lu, Maode Ma, Haiyong Bao

**Affiliations:** 1 School of Electrical and Electronic Engineering, Nanyang Technological University, Singapore 639798, Singapore; qlkong@ntu.edu.sg (Q.K.); emdma@ntu.edu.sg (M.M.); 2 Faculty of Computer Science, University of New Brunswick, Fredericton, NB E3B 5A3, Canada; 3 School of Computer Science and Information Engineering ,Zhejiang Gongshang University, Hangzhou 310018, China; baohy@zjgsu.edu.cn

**Keywords:** range query, location privacy preservation, vehicular sensing

## Abstract

Modern vehicles are equipped with a plethora of on-board sensors and large on-board storage, which enables them to gather and store various local-relevant data. However, the wide application of vehicular sensing has its own challenges, among which location-privacy preservation and data query accuracy are two critical problems. In this paper, we propose a novel range query scheme, which helps the data requester to accurately retrieve the sensed data from the distributive on-board storage in vehicular ad hoc networks (VANETs) with location privacy preservation. The proposed scheme exploits structured scalars to denote the locations of data requesters and vehicles, and achieves the privacy-preserving location matching with the homomorphic Paillier cryptosystem technique. Detailed security analysis shows that the proposed range query scheme can successfully preserve the location privacy of the involved data requesters and vehicles, and protect the confidentiality of the sensed data. In addition, performance evaluations are conducted to show the efficiency of the proposed scheme, in terms of computation delay and communication overhead. Specifically, the computation delay and communication overhead are not dependent on the length of the scalar, and they are only proportional to the number of vehicles.

## 1. Introduction

Nowadays, the fast development of automotive industry and the wide deployment of on-board sensors have created a huge opportunity of vehicular sensing [1]. Other than protecting the normal operations of vehicles, the data generated by on-board sensors (e.g., chemical spill detectors, vibration sensors, and acoustic detectors [2]) can also provide unprecedented spatial-temporal coverage and witness unpredictable incidents with virtually zero investment in the deployment and maintenance of fixed surveillance infrastructures [3]. Meanwhile, since modern vehicles are normally not constrained by energy supply and equipped with adequate on-board storage (up to terabytes), they can keep the continuously harvested data in their on-board storage, which avoids the network congestion caused by the sensed data uploading. Motivated by the practical profits of vehicular sensing, various vehicular sensing based applications have appeared. For example, MobEyes [2] proposes a proactive urban monitoring application with the data collected from cameras and chemical detection sensors, a road surface condition detection application [4] is devised by opportunistically gathering data from vibration and GPS sensors. Meanwhile, to attract sufficient participants to join in the sensing process, an incentive mechanism for mobile crowdsensing has been devised in [5].

However, the wide application of vehicular sensing has met several challenges [6,7,8]. The first challenge is related to the location privacy of data requesters and data uploading vehicles. To retrieve the wanted data from the distributive on-board storage, each data query should specify the corresponding time and location. However, if the location information of a data query is disclosed, it may bring social reputation or economic damage to the querying location. Meanwhile, since vehicles are dynamically moving, their data reports should also be spatial-temporal tagged. The location information of a vehicle can be correlated with certain personal affairs (such as churches and hospitals), or it may reveal the identities of the vehicle owners (such as residences and offices). Without location privacy preservation, data requesters and vehicles are reluctant to issue data queries and upload sensed data, which leads to the under utilization of vehicular sensing. Thus, the location privacy of data requesters and vehicles should be preserved.

The second challenge is related to the accuracy of data query results. Due to the sheer volume of on-board data generation and limited transmission bandwidth, it is impossible for vehicles to upload all the sensed data immediately, and some of the sensed data are maintained in their on-board storage (except those real-time data for applications with stringent delay requirements) [9]. Since vehicles are dynamically and opportunistically moving, the sensory data maintained in their on-board storage captured during a past time period may or may not be generated within the target query area. Moreover, it is highly possible that the queried sensory data are partially generated within the target query area. Thus, it is difficult for the data requester to accurately identify and acquire the wanted data from the on-board storage of the massive and dynamically moving vehicles. However, most of the current secure range query schemes are devised for the outsourced central cloud storage [10,11], which cannot be directly applied to the distributive vehicular storage. Thus, a secure and accurate range query scheme is needed for the distributive on-board storage scenario to fully exploit the potential of vehicular sensing.

In this paper, to overcome the above challenges, we propose a novel privacy-preserving range query scheme from the distributive on-board storage in vehicular ad hoc networks (VANETs) in a practical scenario: acquiring the data harvested by the on-board air pollution sensors within the defined query area, i.e., an industrial area, to monitor the air quality. The proposed scheme exploits the Paillier cryptosystem [12] to preserve the location privacy of data requesters and vehicles, and protect the confidentiality of the sensed data. Specifically, the contributions of this paper are threefold.

First, the proposed scheme structures each multi-dimension scalar in one dimension, where the multi-dimension scalars denote the positions of the data requesters and vehicles in the format of grid cells; meanwhile, the proposed scheme supports secure scalar product computation for location matching. Thus, the location-based data query and data reports can be transmitted to the data server with high efficiency; meanwhile, the data harvested within the target query area can still be identified with location privacy preservation.

Second, since the vehicles are dynamically moving, the average of the sensory data captured during a short-time period may contain both the sensory data generated within and outside the query area. The proposed scheme enables the identification of the number of data reports generated within the target query area, and guarantees the accuracy of the sensory data captured within the target area.

Third, we give detailed security analysis to show that the proposed scheme is secure under the defined security model and achieves the security requirements in terms of location privacy preservation and confidentiality. Meanwhile, we conduct comparative performance evaluation to show that the proposed scheme is more efficient than the existing homomorphic secure scalar product schemes in terms of computational complexity and communication overhead.

The remainder of this paper is organized as follows. We describe the system model, the security requirements, and the design goals in Section 2. We recall the preliminaries and propose our privacy-preserving range query scheme in Section 3, followed by our security analysis and performance evaluations in Section 4 and Section 5, respectively. We show related work in Section 6, and finally conclude our work in Section 7.

## 2. System Model, Security Requirement and Design Goal

### 2.1. System Model

**Figure 1:**
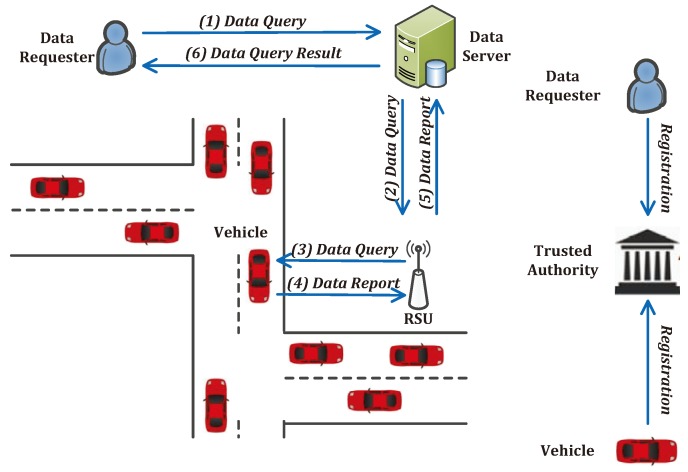
Data query architecture.

In the system model, we present the proposed range query scheme in a practical vehicular sensing application: help the data requester to acquire the data generated by vehicles (data harvested by the on-board air pollution sensors [13]) located within the target query area (an industrial district) during a past short-time period, which can help to investigate the air quality of the given industrial district. The system model consists of five entities, as shown in Figure 1.

- The **data requester** aims to gather the air quality data in a given industrial district from the on-board air pollution sensors. The data requester submits a location-based data query *((1) Data Query)* towards the data server and waits to hear the reply, as shown in Figure 1.
- When the **data server** receives a data query request from a data requester, it forwards the received data query request *((2) Data Query)* to all the vehicles through road side units (RSUs). Meanwhile, the data server receives all the on-board sensed data reports from RSUs, performs data filtering according to the data query, and delivers the data query response towards the data requester *((6) Data Query Result)*, as shown in Figure 1.
- Each **RSU** serves as a gateway between the data server and data uploading vehicles, it helps to broadcast received data queries towards all the vehicles under its coverage *((3) Data Query)* and forward the received on-board sensed data reports towards the data server *((5) Data Report)*, as shown in Figure 1.
- Each **data uploading vehicle** is equipped with the required on-board sensor and enough on-board storage. When a piece of data is harvested by the on-board sensor of a vehicle, it is maintained in the on-board storage with time and location tagged. When a vehicle receives a data query, it first checks whether the queried data is still maintained. If the vehicle owns the queried data, it uploads the location-based sensed data to the nearest RSU*((4) Data Report)*, as shown in Figure 1.
- The **trusted authority** is a trusted and powerful entity, which is mainly responsible for the system bootstrap, key materials management, and the registration of new data requesters and vehicles, as shown in Figure 1.

The wireless connection between a vehicle and an RSU is realized through the IEEE 802.11p standard, a short- to medium-range communication technology operating at 5.85–5.925 GHz band with 3–27 Mbps data rates [14], which is mainly designed for the intelligent transportation systems radio service. The connections between RSUs and the data server, and those between data requesters and the data server, are realized through either wired links or any other links with high bandwidth and low transmission delay.

### 2.2. Security Requirements

In the security model, we consider the trusted authority is fully trusted, while the data server and RSUs are assumed to be honest-but-curious, that is, they follow the protocols, but they may try to infer the generation location and the content of data queries and data reports, which may violate the location privacy and confidentiality. Therefore, to preserve the location privacy of data requesters and vehicles, and protect the confidentiality of each individual sensed data report, the following security requirements should be satisfied:

*Location Privacy*. Protect the location privacy indicates that the location information contained in each data query and data report should be protected [6], and the location information in this context indicates the grid cell scalars, which the data query and data generation locations are mapped to. Even if the data server obtains all the possible data queries and data reports, it cannot identify the grid cell scalar contained in any location-based data query or data report. Location privacy protection also includes that the data uploading vehicles cannot learn the grid cell scalar of the data query, or vice versa. In this way, the location privacy of data requesters and data uploading vehicles can be preserved.

*Confidentiality*. Protect the confidentiality of individual sensed data report means that, even if the data server obtains all the possible on-board sensed data reports, it cannot recover the content of an individual sensed data report [15]. Thus, the individual on-board sensed data can achieve the security requirement of confidentiality .

Note that there may exist other types of attacks such as impersonation, eavesdropping, and violation of data integrity [16]. Since we mainly focus on the privacy-preserving range query from the distributive on-board storage in VANETs, these security threats are beyond our study scope. We also assume that there is no collusion attack in the system, which is in accordance with previous research on secure data query [17].

### 2.3. Design Goals

Under the aforementioned system model and security requirements, our design goal is to develop a privacy-preserving range query scheme from the distributive on-board storage in VANETs. Specifically, the following three objectives should be achieved.

According to the above statement, if the proposed range query scheme does not take security into consideration, the location privacy of data requesters and data uploading vehicles could be threatened. Then, data requesters and vehicles may not be willing to participate in the range query process, and the system cannot properly operate. Therefore, the proposed scheme should achieve the security goal of location privacy.

To accurately reflect the air quality, it is important for data requesters to accurately obtain the data harvested by vehicles in the given query area. Since vehicles are dynamically moving and their positions are opportunistically changing, each received sensed data report may or may not be able to reflect air quality. Thus, to achieve the goal of the high accuracy in air quality monitoring, the data server should accurately filter all the received data reports.

Since vehicles are featured with the fast-moving characteristic, the connections between RSUs and vehicles are relatively short and intermittent, and the communication overhead introduced by the data query and sensed data reports should also be minimized. To extract the desired data from the massive amount of sensory data reports efficiently, the computational complexities brought to data server should also be deliberately evaluated.

## 3. Proposed Scheme

In this section, we propose the privacy-preserving range query scheme from the distributive on-board storage in VANETs, which mainly consists of five parts: preliminaries, system initialization, data query generation, data report generation, and data filtering.

### 3.1. Preliminaries

In our proposed scheme, the Paillier cryptosystem is exploited due to its additive homomorphic property and the homomorphic multiplication property of one plaintext and one ciphertext [12], which has been widely employed in many privacy-preserving data processing applications [15]. Specifically, the Paillier cryptosystem consists of three components: key generation, encryption, and decryption.

- *Key Generation:* Given the security parameter κ, two large prime numbers $p_{1},q_{1}$ are first chosen, where $|p_{1}\left|=\right|q_{1}|=\kappa$. Then, the RSA modulus $n=p_{1}q_{1}$ and $\lambda =lcm(p_{1}-1,q_{1}-1)$ are computed. Define a function $L\left(u\right)=\frac{u-1}{n}$, after choosing a generator $g\in Z_{n^{2}}^{*}$, $\mu ={\left(L\left(g^{\lambda }\;\mathrm{mod}\;n^{2}\right)\right)}^{-1}\;\mathrm{mod}\;n$ is further calculated. Then, the public key is pk=(n,g), and the corresponding private key is sk=(λ,μ).
- *Encryption:* Given a message $m\in Z_{n}$, choose a random number $r\in Z_{n}^{*}$, and the ciphertext can be calculated as $c=E\left(m\right)=g^{m}\cdot r^{n}\;\mathrm{mod}\;n^{2}$.
- *Decryption:* Given the ciphertext $c\in Z_{n^{2}}^{*}$, the corresponding message can be recovered as $m=D\left(c\right)=L\left(c^{\lambda }\;\mathrm{mod}\;n^{2}\right)\cdot \mu \;\mathrm{mod}\;n$.

### 3.2. System Initialization

For the range query system under consideration, we assume a trusted authority, located at the management authority of vehicles and traffics, will bootstrap the whole system.

Given a security parameter κ, the trusted authority selects two large prime numbers $p_{1}$ and $q_{1}$, where $|p_{1}\left|=\right|q_{1}|=\kappa$. The trusted authority also calculates the Paillier cryptosystem’s public key $\left(n=p_{1}q_{1},g\right)$, and the corresponding private key (λ,μ). Moreover, the trusted authority chooses a secure cryptographic hash functions H(), where $H:{\left\{0,1\right\}}^{*}\to Z_{n}^{*}$. In addition, the trusted authority also chooses a random number α. The trusted authority publishes the system parameter as params={n,g,H()}.

**Figure 2:**
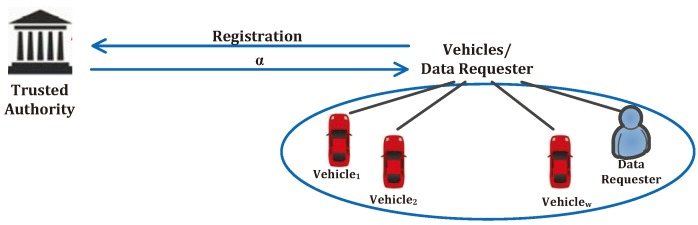
Registration and secret sharing among the data requester and vehicles.

The trusted authority assigns the private key (λ,μ) towards the data server, but the trusted authority does not share α with the data server. During the registration of each data uploading vehicle (*w* vehicles in total) or each data requester, the trusted authority checks its eligibility (whether the vehicle has the on-board air pollution sensors installed) and securely returns α towards it, as shown in Figure 2.

### 3.3. On-Board Data Query Generation

In this subsection, we first describe the query area construction. Then, we identify the data query generation process.

#### 3.3.1. Query Area Construction

**Figure 3:**
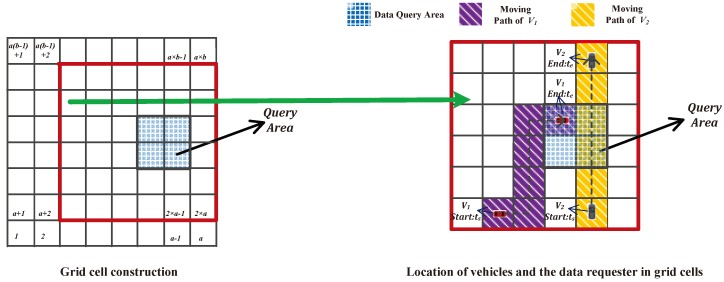
Defined grid cell architecture/locations of vehicles and the data query area in grid cells.

A data requester, the environmental protection agency in this context, aims to monitor the air quality of an industrial district through collecting the data generated by the air pollution sensor (Data_Type), during a past time period (from the starting time $t_{s}$ to the ending time $t_{e}$). We exploit the grid cell system defined in [18] to construct a grid cell architecture, which reflects the locations of the data requester and vehicles: the data requester also specifies a large region contains the target industrial district, which is large enough for the data requester and vehicles to be comfortable with revealing the fact that they are somewhere within this query area, and the bottom-left vertex of the large region is with the bottom-left vertex $\left(x_{0},y_{0}\right)$. The large region is constructed into a grid cell structure, which is divided into k=a×b equal-sized grid cells with the side length of *L*, i.e., L=0.8 km, when we take an industrial area with the coverage of 2.4 km${}^{2}$ as an example [19]. Given location $L_{i}$ with coordinates $\left(x_{i},y_{i}\right)$, the corresponding grid cell identifier can be computed as $\left\lceil \frac{x_{i}-x_{0}}{L}\right\rceil +\left\lfloor \frac{y_{i}-y_{0}}{L}\right\rfloor \cdot a$. As shown in Figure 3, the data query area and the moving trajectory of vehicles are denoted with grid cells.

#### 3.3.2. Data Query Generation

The data requester generates a query area that includes a group of grid cells $I_{d}$, which is overlapped with the target industrial district. Meanwhile, the data requester generates a grid cell scalar $\vec{u}=\left\{u_{1},\ldots ,u_{k}\right\}$ to denote the defined grid cell architecture shown in Figure 3, which is initially set to zero. If $i\in I_{d}$, i.e., grid cell *i* belongs to the target industrial district, we set the corresponding position in the scalar $\vec{u}$ to $u_{i}=1$. Moreover, the data requester selects a number β, which satisfies the condition that $2\cdot k<2^{\beta }$ and $2^{(2k+1)\beta }<n$. In addition, we structure the multi-dimension grid cell scalar to one composite value by calculating $m_{u}=\sum_{i=1}^{k}u_{i}\cdot 2^{i\cdot \beta }$, and generate the ciphertext of $m_{u}$, which is

(1) $$\left\{\begin{array}{c} C_{u,1}=g^{m_{u}+H\left(\alpha ||1||TS\right)}\cdot r_{u,1}^{n}\;\mathrm{mod}\;n^{2},\\ C_{u,2}=g^{m_{u}\cdot H{\left(\alpha ||1||TS\right)}^{-1}+H\left(\alpha ||2||TS\right)}\cdot r_{u,2}^{n}\;\mathrm{mod}\;n^{2}, \end{array}\right.$$

where TS is the current timestamp, and $r_{u,1},r_{u,2}\in Z_{n}^{*}$ are two random numbers.

Finally, the data requester formulates a data request and delivers it to the data server:

(2) $$Request\leftarrow \langle Data\_Type∥x_{0}∥y_{0}∥a∥b∥L∥\beta ∥t_{s}∥t_{e}∥▵t∥C_{u,1}∥C_{u,2}∥TS\rangle ,$$

where ▵t is the on-board sensed data sampling interval.

After receiving Request, the data server further delivers the data request

(3) $$Request_{1}\leftarrow \langle Data\_Type∥x_{0}∥y_{0}∥a∥b∥L∥\beta ∥t_{s}∥t_{e}∥▵t∥TS\rangle$$

towards the data uploading vehicles through RSUs.

### 3.4. Data Report Generation

For each registered data uploading vehicle, the previously sensed data (on the scale of a few past days) and the related information (such as number, time, data type, sensed data, location coordinates, etc.) are maintained in its on-board storage in the format of 〈n,t,Data_Type,data,(x,y)〉. Upon receiving $Request_{1}$, it checks its on-board storage to identify the data in consistent with the data query. If the queried data exists, the vehicle performs the following steps.

The vehicle identifies the on-board air pollution sensor data $\left(data_{1},data_{2},\ldots ,data_{d}\right)$ harvested at the past time points $\left(t_{s},t_{s}+▵t,\ldots ,t_{e}\right)$, where $d=\frac{t_{e}-t_{s}}{▵t}+1$, d<k, ▵t is the sampling interval. Then the vehicle calculates the sum of the sensed data reports, which is denoted as $sum_{v}=\sum_{i=1}^{d}data_{i}$ and it satisfies the condition that $\sum_{v=1}^{w}sum_{v}<n$, where *w* denotes the total number of data uploading vehicles. In addition, the vehicle generates the ciphertext of the on-board sensed data $sum_{v}$:

(4) $$C_{v,0}=g^{sum_{v}+H\left(\alpha ||0||TS\right)}\cdot r_{v,0}^{n}\;\mathrm{mod}\;n^{2},$$

where $r_{v,0}\in Z_{n}^{*}$ is a random number.

Then, the vehicle maps the location coordinates of the on-board sensed data into grid cell architecture shown in Figure 3, and generates a vector $\vec{v}=\left\{v_{1},\ldots ,v_{k}\right\}$ with Algorithm 1.
**Algorithm 1** Vehicle Scalar Generation**Data:** Given location coordinates $\left\{\left(x_{1},y_{1}\right),\left(x_{2},y_{2}\right),\ldots ,\left(x_{d},y_{d}\right)\right\}$ and sets $\vec{v}=\left\{v_{1},\ldots ,v_{k}\right\}=\vec{0}$.**Compute:**
1:**for** i=1:d **do**2: Given location coordinates $L_{i}\left(x_{i},y_{i}\right)$, the vehicle calculates $j=\left\lceil \frac{x_{i}-x_{0}}{L}\right\rceil +\left\lfloor \frac{y_{i}-y_{0}}{L}\right\rfloor \cdot a$;3: **if** j∈{1,2,…,k} **then**4:  Calculates $v_{j}=v_{j}+1$;5: **end if**6:**end for****Output:**
$\vec{v}$

Then, the vehicle structures the multi-dimensional scalar $\vec{v}$ into one dimension : $m_{v}=\sum_{j=1}^{k}v_{j}\cdot 2^{(k+1-j)\cdot \beta }$, and generates the ciphertext

(5) $$\left\{\begin{array}{c} C_{v,1}=g^{m_{v}+H{\left(\alpha ||1||TS\right)}^{-1}}\cdot r_{v,1}^{n}\;\mathrm{mod}\;n^{2},\\ C_{v,2}=g^{m_{v}\cdot H\left(\alpha ||1||TS\right)-H\left(\alpha ||2||TS\right)-\eta_{v}}\cdot r_{v,2}^{n}\;\mathrm{mod}\;n^{2}, \end{array}\right.$$

where $r_{v,1},r_{v,2}\in Z_{n}^{*}$ are two random numbers, and $\eta_{v}=\eta_{v,1}\cdot 2^{\beta }+\ldots +\eta_{v,k}\cdot 2^{k\cdot \beta }+\eta_{v,k+1}\cdot 2^{(k+2)\cdot \beta }$, such that $\eta_{v,i}$ is a random number that satisfies the condition: $\eta_{v,i}+k<2^{\beta }$ when i∈{1,…,k} and $\eta_{v,k+1}\in \left[1,k\cdot 2^{(k-2)\cdot \beta }\right)$.

Finally, the vehicle formulates the Data_Report and delivers it to its nearest RSU:

(6) $$Data\_Report\leftarrow \langle C_{v,0}∥C_{v,1}∥C_{v,2}\rangle .$$

### 3.5. Privacy Preserving Data Filtering

Upon receiving all the data reports, each RSU forwards all the collected on-board sensed data reports to the data server, and the data server filters within all the received data reports to identify the on-board sensed data reports generated within the industrial district.

The data server first computes the scalar product of $\vec{u}$ and $\vec{v}$ to identify the data reports generated within the industrial district, based on the idea that the data uploading vehicle must share at least one grid cell with the industrial district. Then, the data server decrypts $C_{u,1}$ with its private key sk and recovers the value of $m_{u}+H\left(\alpha ||1||TS\right)$. Meanwhile, the data server also computes

(7) $$\begin{array}{c} C_{u,v}=\frac{{\left(C_{v,1}\right)}^{m_{u}+H\left(\alpha ||1||TS\right)}}{C_{u,2}\cdot C_{v,2}}\;\mathrm{mod}\;n^{2}\\ =\frac{{\left(g^{m_{v}+H{\left(\alpha ||1||TS\right)}^{-1}}\cdot r_{v,1}^{n}\right)}^{m_{u}+H\left(\alpha ||1||TS\right)}}{g^{m_{u}H{\left(\alpha ||1||TS\right)}^{-1}+m_{v}H\left(\alpha ||1||TS\right)-\eta_{v}}\cdot r_{u,2}^{n}\cdot r_{v,2}^{n}}\;\mathrm{mod}\;n^{2}\\ =g^{m_{u}m_{v}+\eta_{v}+1}\cdot (\frac{r_{v,1}^{m_{u}+H\left(\alpha ||1||TS\right)}}{r_{u,2}\cdot r_{v,2}}{)}^{n}\;\mathrm{mod}\;n^{2}. \end{array}$$

Based on $C_{u,v}$, the data server recovers the value of $s_{u,v}=m_{u}m_{v}+\eta_{v}+1$ with the private key sk. With $s_{u,v}$, the data server can calculate the scalar product of $\vec{u}\cdot \vec{v}$,

(8) $$\;k_{v}=\vec{u}\cdot \vec{v}=\frac{\left(s_{u,v}\;\mathrm{mod}\;2^{(k+2)\cdot \beta }\right)-\left(s_{u,v}\;\mathrm{mod}\;2^{(k+1)\cdot \beta }\right)}{2^{(k+1)\cdot \beta }},$$

where $k_{v}$ indicates the pieces of sensed data generated within the industrial district. If $k_{v}=\vec{u}\cdot \vec{v}\geq 1$, at least one piece of sensed data is generated in industrial district, and the vehicle becomes a member of the group $N_{in}$; otherwise, all the data reports are generated out of the industrial district, and the vehicle becomes a member of the group $N_{out}$, such that $|N_{in}\left|+\right|N_{out}|\leq w$. Finally, the data server calculates $K_{in}=\sum_{v\in N_{in}}k_{v}$.

*Correctness.* The correctness of Equation (8) can be clearly illustrated with the following equation:

(9) $$\;\begin{array}{c} s_{u,v}=m_{u}m_{v}+\eta_{v}+1\\ =u_{1}v_{1}\cdot 2^{(k+1)\beta }+u_{2}v_{1}\cdot 2^{(k+2)\beta }+\ldots +u_{k}v_{1}\cdot 2^{2k\beta }\\ +u_{1}v_{2}\cdot 2^{k\beta }\;\;\;\;\;+u_{2}v_{2}\cdot 2^{(k+1)\beta }+\ldots +u_{k}v_{2}\cdot 2^{(2k-1)\beta }\\ +\;\;\ldots \;\;\\ +u_{1}v_{k}\cdot 2^{2\beta }\;\;\;\;\;+u_{2}v_{k}\cdot 2^{3\beta }\;\;\;\;\;+\ldots +u_{k}v_{k}\cdot 2^{(k+1)\beta }\\ +\eta_{v,k+1}2^{(k+2)\beta }+\eta_{v,k}2^{k\beta }\;\;\;\;\;\;\;\;+\ldots +\eta_{v,1}2^{\beta }+1\\ =(\sum_{i=1,i<j}^{k}u_{i}v_{j}\cdot 2^{(k+1+i-j)\beta }+\eta_{v,k}2^{k\beta }+\ldots +\eta_{v,1}2^{\beta }+1)\\ +(\sum_{i=1,i>j}^{k}u_{i}v_{j}\cdot 2^{(k+1+i-j)\beta }+\eta_{v,k+1}2^{(k+2)\beta })\\ +(\sum_{i=1,i=j}^{k}u_{i}v_{j}\cdot 2^{(k+1)\beta }). \end{array}$$

As shown in Equation (9), $s_{u,v}$ consists of three parts: (i) when i<j, $\left(\sum_{i=1,i<j}^{k}u_{i}v_{j}\cdot 2^{(k+1+i-j)\beta }+\eta_{v,k}2^{k\beta }+\ldots +\eta_{v,1}2^{\beta }+1\right)<2^{(k+1)\beta }$; (ii) when i>j, $\left(\sum_{i=1,i>j}^{k}u_{i}v_{j}\cdot 2^{(i-j-1)\beta }+\eta_{v,k+1}\right)\cdot 2^{(k+2)\beta }$; and (iii) when i=j, $\left(\sum_{i=1,i=j}^{k}u_{i}v_{j}\cdot 2^{(k+1)\beta }\right)$. Thus, with Equation (8), $\vec{u}\cdot \vec{v}$ can be computed.

### 3.6. Data Report Aggregation

The data server first aggregates the group of sensed data generated outside the industrial district $N_{out}$, which is

(10) $$\begin{array}{c} C_{out}=\prod_{v\in N_{out}}C_{v,0}\;\mathrm{mod}\;n^{2}\\ \;\;\;\;\;=g^{\sum_{v\in N_{out}}(sum_{v}+H(\alpha ||0||TS))}\cdot (\prod_{v\in N_{out}}r_{v,0}{)}^{n}\;\mathrm{mod}\;n^{2}. \end{array}$$

Then, the data server decrypts $C_{out}$ with the private key sk, and calculates the average of the sensed data generated outside the industrial district, which is $S_{out}=\sum_{v\in N_{out}}(sum_{v}+H(\alpha ||0||TS))$. Meanwhile, the data server aggregates the sensed data within the group $N_{in}$, which is

(11) $$\begin{array}{c} C_{in}=\prod_{v\in N_{in}}C_{v,0}\;\mathrm{mod}\;n^{2}\\ \;\;\;\;\;=g^{\sum_{v\in N_{in}}(sum_{v}+H(\alpha ||0||TS))}\cdot (\prod_{v\in N_{in}}r_{v,0}{)}^{n}\;\mathrm{mod}\;n^{2}. \end{array}$$

In addition, the data server decrypts $S_{in}=\sum_{v\in N_{in}}(sum_{v}+H(\alpha ||0||TS))$ with the private key sk; meanwhile, the data server delivers $S_{in}$, $S_{out}$, $K_{in}$, $|N_{in}|$ and $|N_{out}|$ towards the data requester.

After receiving $S_{in}$, $S_{out}$, $K_{in}$, $|N_{in}|$ and $|N_{out}|$, the data requester first calculates the average of the sensed data outside the industrial district, which is

(12) $$Ave_{out}=\frac{(S_{out}-|N_{out}|\cdot H(\alpha ||0||TS))\;\mathrm{mod}\;n}{d\cdot |N_{out}|}.$$

Then, the data requester calculates the average of the sensed data inside the industrial district, which is

(13) $$Ave_{in}=\frac{(S_{in}-\left(\right|N_{in}|\cdot d-K_{in})\cdot Ave_{out}-|N_{in}|\cdot H(\alpha ||0||TS))\;\mathrm{mod}\;n}{K_{in}}.$$

## 4. Security Analysis

Following the earlier discussed security requirements, we analyze the security properties of the proposed privacy-preserving range query scheme in this section. Specifically, our analysis will focus on how the proposed scheme can achieve the location privacy preservation of data requesters and data uploading vehicles, and protect the confidentiality of the individual on-board sensed data.

*The proposed range query scheme can preserve the location privacy of data requester and data uploading vehicles.* In the proposed scheme, the data requester first identifies a large region to construct the grid cell structure, which contains the industrial district. Based on the constructed grid cell structure, nothing could be learned besides the fact that the data query occupies one or a few grid cells in the defined grid cell structure. Since the location of the data requester and the vehicles are mapped to the constructed grid cell architecture, protecting the grid cell scalar of the data requester and the vehicles means preserving the location privacy. The following two paragraphs illustrate how the proposed scheme can protect the grid cell scalar during the secure scalar product computation process.

In the proposed scheme, the data requester’s multi-dimensional grid cell scalar $\vec{u}$ is structured into one dimension $m_{u}$, and then encrypted by the public key of the data server pk to generate $\left(C_{u,1},C_{u,2}\right)$. After receiving $\left(C_{u,1},C_{u,2}\right)$, the data server decrypts $C_{u,1}$ with its private key sk, and obtains $m_{u}+H\left(\alpha ||1||TS\right)$. To prevent the data server from recovering the value of $m_{u}$, H(α||1||TS) and H(α||2||TS) are introduced in $\left(C_{u,1},C_{u,2}\right)$. Since the secret α is only shared between the data requester and data uploading vehicles and the defined security model does not take collusion attack into consideration, the data server cannot recover the value of $m_{u}$. As $\left(C_{u,1},C_{u,2}\right)$ are valid ciphertexts of the Paillier cryptosystem, which is known to be semantically secure against the chosen plaintext attack, and the composite grid cell scalar contained in $\left(C_{u,1},C_{u,2}\right)$ is also semantically secure. Meanwhile, the locations of data uploading vehicles are also encrypted by the public key of the data server and protected by the secret α. Since only the data server with the private key sk can decrypt $\left(C_{u,1},C_{u,2}\right)$ and $\left(C_{v,1},C_{v,2}\right)$, and the data server cannot recover the value of $m_{u}$ and $m_{v}$ with the decrypted results, the composite grid cell scalars of the data requester and vehicles can be protected during transmission.

Due to the homomorphic property of the Paillier cryptosystem, the data server first computes ${\left(C_{v,1}\right)}^{m_{u}+H\left(\alpha ||1||TS\right)}$ and then recovers the value of $s_{u,v}=m_{u}\cdot m_{v}+\eta_{v}+1$ through decrypting $\frac{{\left(C_{v,1}\right)}^{m_{u}+H\left(\alpha ||1||TS\right)}}{C_{u,2}\cdot C_{v,2}}$. To avoid the exhaustive attack against $m_{u}\cdot m_{v}$, a random number $\eta_{v}$ is also included in $s_{u,v}$, but the data server can still obtain the scalar product of $\vec{u}\cdot \vec{v}$ by computing $\frac{\left(s_{u,v}\;\mathrm{mod}\;2^{(k+2)\beta }\right)-\left(s_{u,v}\;\mathrm{mod}\;2^{(k+1)\beta }\right)}{2^{(k+1)\beta }}$. In this way, the content of each composite grid cell scalar can still be protected. Meanwhile, the location privacy of the data requester and data uploading vehicles can be preserved during the secure scalar product computation.

*The individual on-board sensed data report is confidential in the proposed scheme.* In the proposed scheme, each vehicle’s on-board sensed data report is formed as $C_{v,0}=g^{sum_{v}+H\left(\alpha ||0||TS\right)}\cdot {\left(r_{v,0}\right)}^{n}\;\mathrm{mod}\;n^{2}$. Since $C_{v,0}$ is a valid ciphertext of the Paillier cryptosystem, the sensed data $sum_{v}$ in $C_{v,0}$ is also semantically secure. After decrypting $C_{v,0}$ with sk, the data server still cannot recover the value of $sum_{v}+H\left(\alpha ||0||TS\right)$, since α is a secret shared between the data requester and vehicles. In addition, the data server aggregates all the sensed data within the group $N_{in}$ and the group $N_{out}$, respectively, and delivers the aggregated results $S_{in}$ and $S_{out}$ to the data requester. Without α, the data server also cannot learn the aggregation of the sensed data reports $sum_{v}$, and the confidentiality of the aggregation results can be achieved. Furthermore, the data requester can only obtain the aggregated results contained in $S_{in}$ and $S_{out}$, but it cannot recover the value of each individual sensed data. Therefore, the confidentiality of individual sensed data can also be protected.

## 5. Performance Evaluation

In this section, we describe the parameter setup, and evaluate the performance of the proposed scheme in terms of computation complexity and communication overhead.

### 5.1. Parameter Setup

We conduct the experiments with the Java Paillier Library [20] on a desktop with 3.40 GHz processor and 8.00 GB memory to study the operation costs. The experimental result shows that the cost of a single exponentiation operation in $Z_{n^{2}}^{*}$ ($|n^{2}|=2048$) is $C_{e}=8.82$ ms. The proposed scheme integrates all the elements in a scalar to one composite value, which requires us to choose the proper parameter β and the length of the scalar *k*. Given |n|=1024, the maximum value of $\beta_{max}$ is 7 and the maximum length of the scalar is $k_{max}=63$, which satisfies the condition that $2\cdot k_{max}<2^{\beta_{max}}$ and $\left(2\cdot k_{max}+1\right)\cdot \beta_{max}<\left|n\right|$.

We compare the proposed scheme with the traditional homomorphic secure scalar product when Paillier encryption is exploited, i.e., each element in a scalar is encrypted separately at the data requester side, and then transmitted to the data server, i.e., the ciphertext of the data requester are generated as $C_{u,1,i},C_{u,2,i},i=1,2,\ldots ,k$. At the vehicle side, each element in a scalar is also encrypted and delivered individually, i.e., the ciphertext of each vehicle are generated as $C_{v,1,i},C_{v,2,i},i=1,2,\ldots ,k$. At the data server side, the product of two corresponding elements in the two scalars are also computed individually, $C_{u,v,i}=\frac{{\left(C_{v,1,i}\right)}^{u_{i}+H\left(\alpha ||1||TS\right)}}{C_{u,2,i}\cdot C_{v,2,i}}$, and then aggregated to $C_{u,v}=\prod_{i=1}^{k}C_{u,v,i}$. Finally, the aggregated results are decrypted to derive the final results.

### 5.2. Computation Complexity

In the proposed scheme, when a data requester generates a data query, it requires 4 exponentiation operations in $Z_{n^{2}}^{*}$ to generate $C_{u,1}\left|\right|C_{u,2}$. Note that the computation of $2^{i\cdot \beta },i\in \left\{1,2,\ldots ,k\right\}$ can be conducted in the setup phase, and multiplication operation in $Z_{n^{2}}^{*}$ is negligible in comparison with the exponentiation operation in $Z_{n^{2}}^{*}$. In the traditional scheme, the data requester needs to generate the ciphertext $C_{u,i,1}\left|\right|C_{u,i,2}$ for each element in the scalar separately. For a scalar with the length of *k*, the data requester needs to consume 4×k exponentiation operations in $Z_{n^{2}}^{*}$ to generate the ciphertext. In the proposed scheme, when a vehicle receives a data query, it generates an encrypted location-based data report, which requires 6 exponentiation operations in $Z_{n^{2}}^{*}$. However, for the traditional scheme, each vehicle needs to consume 2+4×k exponentiation operations to generate one data report.

After receiving all the data reports from *w* vehicles, the data server should compute the scalar product of the data requester and each vehicle, to identify whether the on-board data report is harvested within the industrial district. The data server takes one exponentiation operation in $Z_{n^{2}}^{*}$ to recover the value of $m_{u}+H\left(\alpha ||1||TS\right)$ by Paillier decryption, and it also consumes two exponentiation operations in $Z_{n^{2}}^{*}$ to obtain the value of $m_{u}\cdot m_{v}+1$. Since the multiplication operation in $Z_{n^{2}}^{*}$ is considered negligible in comparison to the exponentiation operation in $Z_{n^{2}}^{*}$, the computation cost of aggregation is negligible, and it takes two exponentiation operations in $Z_{n^{2}}^{*}$ for the Paillier decryption to recover the aggregated results. For the traditional scheme, it takes 3×k exponentiation operations in $Z_{n^{2}}^{*}$ to recover the scalar product and costs two exponentiation operations to obtain the aggregated results.

Thus, in the proposed scheme, totally for the data requester, vehicle, and the data server, the computation cost will be $4\times C_{e}$, $6\times C_{e}$, and $\left(3\times w+2\right)\times C_{e}$. For the traditional scheme, the computation cost for the data requester, vehicle, and data server are $4\times k\times C_{e}$, $\left(2+4\times k\right)\times C_{e}$, and $\left(3\times k\times w+2\right)\times C_{e}$, respectively, since each element in a scalar are encrypted and processed independently and the computation complexity is proportional to the length of the scalar.

**Figure 4:**
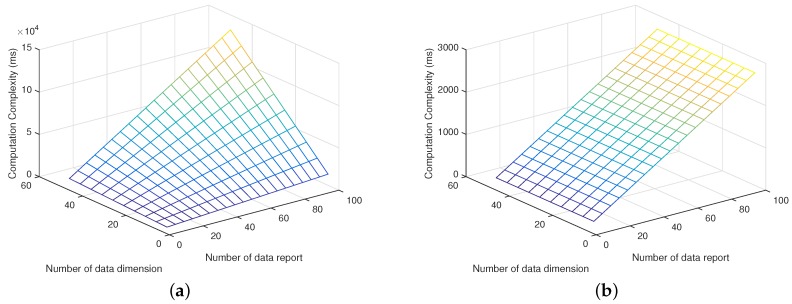
Computation complexity of data server. (**a**) Computation complexity of the data server with the traditional scheme; (**b**) Computation complexity of the data server with the proposed scheme.

**Figure 5:**
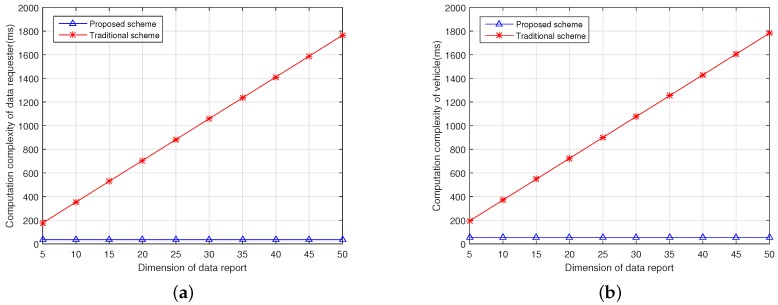
Comparison of the computation complexity of the data requester and vehicle. (**a**) Computation complexity of the data requester; (**b**) Computation complexity of one data uploading vehicle.

Figure 4a,b show the computation complexity of the data server with both schemes in terms of the scalar length and the number of vehicles. Simulation results show that the proposed scheme greatly reduces the computation complexity of the data server in comparison with the traditional scheme. As shown in Figure 4a, the computation complexity increases with both scalar length and the number of vehicles in the traditional scheme, while in the proposed scheme, the computation complexity only increases with the number of vehicles as shown in Figure 4b. Meanwhile, Figure 5a,b present the computation complexity of the data requester and vehicle in terms of the scalar length, and compare the proposed scheme with the traditional scheme, respectively. Figure 4 and Figure 5 indicate that the proposed scheme can achieve lower computation complexity compared to the traditional scheme.

### 5.3. Communication Overhead

We evaluate the communication overhead that introduced the data query, which is sent from the data query towards the data server, and the data report sent from the vehicles towards the data server, since the data query sent from the data server towards the RSU and the data query response sent from the data server do not involve the transmission of ciphertexts in the proposed scheme.

**Figure 6:**
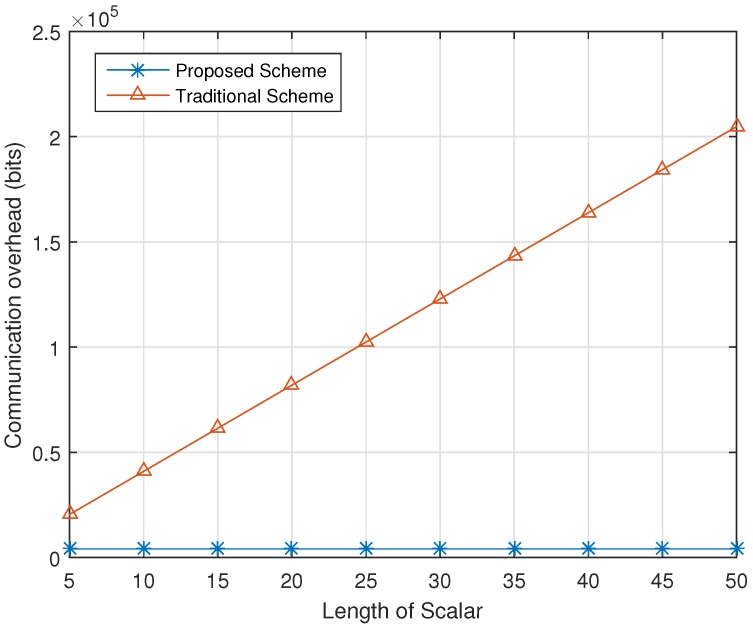
Communication overhead from the data requester to the data server.

We first consider the data requester to data server communication, where the data requester generates a location-based data query and delivers the data query to the data server. The location-based query is in the format of $C_{u,1}∥C_{u,2}$, and its size is 2048×2 bits, if we choose 1024-bit *n*. If the traditional homomorphic scalar product protocol is adopted, the corresponding communication overhead is 2048×2×k bits, when there are *k* partitioned grid cells. In Figure 6, we plot the data requester to data server communication overhead in terms of the scalar length, and compare the proposed scheme to the traditional scheme, which shows that the proposed scheme reduces the communication overhead.

**Figure 7:**
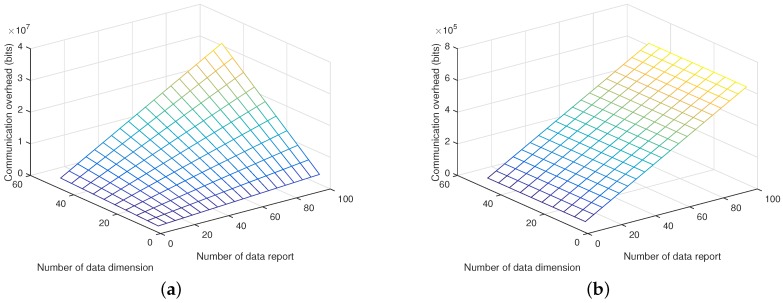
Vehicles to data server communication overhead. (**a**) Communication overhead of vehicles during data uploading with the traditional scheme; (**b**) Communication overhead of vehicles during data uploading with the proposed scheme.

During the vehicle to data server communication, each vehicle generates a location-based data report and delivers the data report to the data server via the RSU. The data report is in the format of $C_{u,0}∥C_{u,1}∥C_{u,2}$, and its size is 2048×3 bits. The data server collects *w* data reports from the total *w* vehicles, the communication overhead between the vehicles and the data server is 2048×3×w bits. For the traditional homomorphic scalar product protocol, the communication between the vehicles and the data server is 2048×3×w×k bits, when the scalar length is *k*. Figure 7a,b plot the communication overhead introduced by vehicles of both schemes in terms of the scalar length and the number of vehicles. Simulation results show that the proposed scheme greatly reduces the vehicle to data server communication overhead in comparison with the traditional scheme. As shown in Figure 7a, the vehicle to data server communication overhead increases with both scalar length and the number of vehicles in the traditional scheme, while in the proposed scheme, vehicle to data server communication overhead only increases with the number of vehicles as shown in Figure 7b.

## 6. Related Works

In this section, we briefly review some of the existing related schemes in secure scalar product computation and secure location-based query schemes.

### 6.1. Secure Scalar Product Computation

Research on secure scalar product computation has been focused on performing privacy-preserving profile matching [21,22,23], and secure multi-party computation [24,25]. A fine-grained privacy-preserving profile matching scheme is proposed with the Paillier homomorphic cryptosystem in [22], which enables two users to measure the level of similarity in their fine-grained personal files. An efficient privacy-preserving binary scalar product computation protocol is proposed in [23], which computes the similarity in symptom characters. A secure multi-party computation algorithm is proposed in [25] with the BGN homomorphic encryption technique, which allows the cloud server to execute secure scalar product and addition operations without the data content disclosure.

However, in the above schemes, each element in a scalar needs to be encrypted separately, which leads to the result that the size of the ciphertexts is proportional to the length to the scalar. In our proposed scheme, the elements in a given scalar is first structured to one-dimension and then encrypted, which is highly efficient in terms of communication and computation overhead.

### 6.2. Secure Location-Based Query

There mainly exist three types of secure location-based data query schemes. The first type of solutions are through pseudonyms [15,26]. However, the pseudonym-based solutions are based on the idea of disrupting the connection between the identities of vehicles and their locations, and the locations of the data queries can still be disclosed [11]. The second type of solutions are through location cloaking [27,28], and the identification of the exact location of each participant can be prevented by introducing uncertainty or error into location information. K-Anonymity is a commonly used technique in location cloaking, which preserves the location privacy of one user through hiding one user among a group of *K* users [29]. Even though location cloaking can achieve a satisfactory level of location privacy preservation, it may not be able to guarantee that there are enough users in the neighborhood and not be able to achieve high accuracy in their query results [30]. The third type of solutions rely on the cryptographic techniques, which can effectively preserve the accuracy of the data query results [31,32]. Various privacy-preserving data query schemes are proposed, such as range query [33]. An efficient spatial range query solution is proposed in [11], which permits data queries over encrypted location based data. The authors in [30] propose a hybrid approach based on location cloaking and the additive homomorphic encryption, which achieves both efficiency and privacy preservation. A spatial range query scheme over ciphertext is proposed in [34], which achieves the location privacy of the user’s query and the location-based service confidentiality.

However, these schemes are based on the scenario of the outsourced central cloud storage data query and introduce heavy cryptographic operations, which also can not be applicable to the dynamically moving and distributive on-board storage. In this paper, we propose an efficient privacy-preserving location-based data query scheme, which can be applied to the scenario of the distributive vehicular on-board storage.

Based on the above analysis, in this paper, we propose an efficient location-based vehicular on-board sensory data querying scheme, which greatly reduces the network complexity and preserve the location privacy of both the data requester and vehicles.

## 7. Conclusions

In this paper, we have proposed a privacy-preserving range query scheme from the distributive on-board storage in VANETs, which achieves the secure scalar product computation with the homomorphic Paillier cryptosystem. The proposed scheme structures the multi-dimensional scalar into one dimension, identifies the data harvested within the industrial district, and computes the aggregation results of the identified sensed data. Security analysis has been conducted to demonstrate its security properties, in terms of location privacy preservation and confidentiality. Performance evaluations have also been done, which indicates that the proposed scheme can significantly reduce the computation complexity and communication overhead. For future work, we will take the possible behaviors of the collusion attack into consideration and design new solutions to resist such attacks.
